# Recreational Skiing- and Snowboarding-Related Extremity Injuries: A Five-Year Tertiary Trauma Center Cohort

**DOI:** 10.7759/cureus.42688

**Published:** 2023-07-30

**Authors:** İzzet Özay Subaşı, Volkan Gür

**Affiliations:** 1 Orthopedics and Traumatology, Erzincan University Faculty of Medicine, Erzincan, TUR

**Keywords:** extremities, injury, winter sports, recreational snowboard, recreational ski

## Abstract

Introduction: Skiing and snowboarding are widely recognized winter sports with a notable risk of sports-related injuries. Comprehensive knowledge of the injuries sustained during skiing and snowboarding is imperative for preventative strategies and for understanding the injury patterns and epidemiological characteristics from surgeons' standpoint, particularly orthopedic surgeons, given that all anatomical regions and systems are susceptible to harm when engaging in these activities.

Objective: Our research aims to investigate the injury patterns and epidemiological characteristics of individuals seeking medical attention at Erzincan University Faculty of Medicine Orthopedics and Traumatology Clinic, Erzincan, Turkey, after experiencing recreational skiing or snowboarding injuries.

Methods: During the period spanning from 2018 to 2023, a retrospective analysis was conducted on patients aged between 18 to 65 years who had sustained isolated extremity injuries while participating in recreational winter sports at the Ergan Mountain Ski Center. The causes of injuries were categorized into two types (skiing and snowboarding) based on the subjective description of the injury scene. The study employed the χ2 test for categorical variables and Student's t-test for continuous variables to conduct subgroup comparisons.

Results: The study results revealed that most patients, precisely 53.2%, sustained injuries in their lower extremities, whereas 46.8% sustained injuries in their upper extremities. The most frequently injured area of the patients presenting with skiing injury was the region around the knee (31.2%), followed by around the shoulder (21.6%) and the foot and ankle (14.9%). The anatomical region most injured among patients who sustained snowboarding injuries was the hand and wrist, accounting for 23.9% of cases.

Conclusion: Skiing and snowboarding injuries vary anatomically. While individuals with limited experience are at a higher risk of sustaining injuries, no statistically significant disparity was observed regarding the specific body parts affected among individuals of varying skill levels. Recognizing these sports-related injuries, which are growing in young people, will encourage the use of personal protective equipment to avoid injuries, assure ski resorts' environmental safety, and reduce sports-related morbidity.

## Introduction

Recreative skiing and snowboarding have gained popularity as winter activities in Turkey. Skiing and snowboarding are widely recognized winter sports with a notable risk of sports-related injuries. Despite demographic, equipment, and skill variations between sports, both entail the potential for traumatic injuries, which may lead to physical, psychological, and financial challenges in the short and long terms [1]. According to reported statistics, such injuries range from 0.5 to 1.35 per 1000 skier/snowboarder days among individuals who engage in these activities recreationally [2-4]. Improving the understanding of injuries among individuals involved in these recreational activities is paramount to facilitating efforts to prevent injuries, thereby reducing the frequency, intensity, and impact of such incidents.

Numerous studies have documented variations in injury patterns among individuals who ski and snowboard [5-7]. Significant distinctions exist between snowboarding and skiing equipment and how riders execute their techniques. In contrast to skiers, who orient themselves in a forward-facing position on their skis, utilize poles, and wear hard-shelled boots equipped with releasable bindings, snowboarders assume a sideways stance on a singular board, forgo the use of poles, and typically wear soft boots with non-releasable bindings [8]. Consequently, the injury patterns exhibited in these two activities will also vary due to the dissimilarities in the movement and fall patterns. Comprehensive knowledge of the injuries incurred during skiing and snowboarding is imperative for orthopedic surgeons, given that all anatomical regions and systems are susceptible to harm when engaging in these activities.

Ergan Mountain (Erzincan, Turkey) is a winter sports destination that offers a suitable environment for winter sports activities for a period of 90-120 days annually. It boasts an impressive infrastructure that can accommodate up to 3450 individuals per hour and features a track length of 6967 meters. It is also located approximately 13 kilometers from our hospital, which is classified as a tertiary trauma center. Our research aims to investigate the injury patterns and epidemiological characteristics of individuals who sought medical attention at our tertiary trauma center, situated close to Ergan Mountain, a prominent winter sports destination in our country, subsequent to experiencing recreational skiing or snowboarding injuries.

## Materials and methods

The study was conducted at Erzincan University Faculty of Medicine Orthopedics and Traumatology Clinic, Erzincan, Turkey. As it involved human research participants, approval was received from Ataturk University Faculty of Medicine Clinical Research Ethics Committee (approval no. B.30.2.ATA.0.01.00/424). Owing to the study's epidemiological nature and to ensure anonymity, the requirement for informed consent was waived. During the period spanning from 2018 to 2023, a retrospective analysis was conducted on patients aged between 18 to 65 years who had sustained isolated extremity injuries while participating in recreational winter sports at the Ergan Mountain Ski Center. The patients were identified through the hospital database system, specifically from those who had sought medical attention at our hospital's emergency department or orthopedics and traumatology outpatient clinic after their injury. The study excluded patients who had sustained injuries beyond extremity injuries, those whose data were incomplete, and those who received further treatment and follow-up at a different medical facility after their initial admission to the emergency department.

The study participants were categorized into two distinct cohorts based on their respective injuries sustained during skiing and snowboarding activities, considering the specific activity in which they were engaged at the time of injury. In addition to demographic data, such as patients' gender and age, our study also documented variables, including the skill level, injury date, and specific body part affected. The patients were categorized based on their self-reported duration of involvement in skiing and snowboarding. The categories included beginner (first season), intermediate (one to five years), advanced (five to 10 years), and expert (≥10 years). The date of the injury was categorized based on the Turkish government's holiday schedule, distinguishing between weekdays and weekends/holidays. The causes of injuries were categorized into two types based on the subjective description of the injury scene. The first type is related to incidents that do not involve others, such as falling or crashing without any physical contact with other individuals. The second type pertains to incidents that involve two or more people, such as crashes that result in physical contact with others. The injured extremities were classified based on the anatomical body regions, which consisted of six categories: 1) the region around the shoulder; 2) the region around the elbow; 3) the hand and wrist region; 4) the pelvis, hip, and thigh region; 5) the region around the knee; and 6) the foot and ankle region. The assessment of patients who have sustained injuries in multiple regions of the extremities was conducted by trauma specialists, who focused their evaluation on the injuries of greater severity.

The statistical analyses were conducted using IBM SPSS Statistics for Windows, Version 24 (Released 2016; IBM Corp., Armonk, New York, United States). The study's continuous variables were reported using the mean and standard deviation, whereas categorical data were presented using frequency counts and percentages. The study employed the χ2 test for categorical variables and Student's t-test for continuous variables to conduct subgroup comparisons. When data were absent, the available case was examined to ascertain the disparity between groups. P<0.05 was considered to indicate statistical significance.

## Results

The present study comprised 304 out of 335 patients admitted to our hospital due to skiing or snowboarding injuries from 2018 to 2023. Of the total number of patients, 23 individuals were found to have sustained injuries to their head, face, thorax, or back and were excluded. However, it is essential to note that eight patients were excluded from the study due to incomplete data accessibility or ongoing treatment at an external medical facility.

Among the patients included in the study group, 34% (104/304) were female. Snowboarders represented 31.3% (96/304) of the patient cohort. The mean age of the patients was 26.4 ± 9.1 in women and 29.3 ±10.3 in men, and no statistically significant difference was found (p=0.09). Injuries that did not involve other skiers (e.g., collision) accounted for 74.6% of all accidents. In our study, 46.7% of the patients received weekday applications to our clinic. Skiers tended to be older than snowboarders, with mean ages of 31.7 ± 11.3 and 29.3 ± 10.3, respectively (p<0.05). Table 1 shows the demographics, injury mechanism, and overall count of patients observed throughout the study.

**Table 1:** Demographics and injury mechanisms of the patients.

	Skiing	Snowboarding
Gender		
Female	79 (37.9%)	25 (26.1%)
Male	133 (62.1%)	71 (73.9%)
Age	31.79±11.39	29.34±10.32
Injury mechanism		
Collusion	59 (28.3%)	18 (18.8%)
Non-collusion	149 (71.6%)	78 (81.2%)
Injury time		
Weekday	97 (46.6%)	45 (46.9%)
Weekend	111 (53.4%)	51 (53.1%)

Of the patients included in the study group, 24.9% were at the beginner level, 35% at the intermediate level, 25.3% at the advanced level, and 14.8% at the expert level. It was observed that around the knee was the most injured area across all patient groups, with a frequency of 87 out of 308 patients (28.6%). At the expert level, the extremity area with the least injury was observed to be around the elbow in the patients, accounting for 11.1% of cases. However, at all other levels except the expert level, the pelvis/hip/thigh region exhibited the least extent of injury. Hip/thigh/pelvis and elbow region injuries were the least detected injuries (8.6% and 10.5%, respectively). There was no significant difference between the self-reported skill levels of the patients and the percentage of injury; however, the majority of the patients in our study were at the beginner or pre-intermediate level. Table 2 shows the regions of the extremities that have been injured categorized according to the patients' respective levels.

**Table 2:** Injured extremity localizations and self-reported skill levels of the patients.

	Beginner (n, %)	Intermediate (n, %)	Advance (n, %)	Expert (n, %)
Shoulder	17 (5.6%)	24 (7.9%)	17 (5.6%)	9 (3%)
Elbow	8 (2.6%)	11 (3.6%)	8 (2.6%)	5 (1.6%)
Hand/wrist	11 (3.6%)	16 (5.3%)	10 (3.3%)	7 (2.3%)
Hip/thigh/pelvis	5 (1.6%)	9 (3%)	6 (2%)	6 (2%)
Knee	22 (7.2%)	30 (9.9%)	24 (7.9%)	11 (3.6%)
Foot/ankle	13 (4.3%)	16 (5.3%)	12 (3.9%)	7 (2.3%)

The study revealed that most patients, precisely 53.2%, experienced injuries in their lower extremities, whereas 46.8% suffered injuries in their upper extremities. The most frequently injured area of the patients presenting with skiing injuries was the region around the knee (31.3%), followed by around the shoulder (21.6%) and the foot and ankle (14.9%). The anatomical region most injured among patients who experienced snowboarding injuries was the hand and wrist, accounting for 23.9% of cases. This was followed by injuries around the shoulder (22.9%) and knee (21.8%). Table 3 displays the injured extremities regions of the patients who participated in our study. 

**Table 3:** Injured extremity regions of skiers (n=208) and snowboarders (n=96).

	Skiing (n, %)	Snowboarding (n, %)
Shoulder	45 (21.6%)	22 (22.9%)
Elbow	26 (12.5%)	6 (6.3%)
Hand/wrist	21 (10.1%)	23 (23.9%)
Hip/thigh/pelvis	20 (9.6%)	6 (6.3%)
Knee	65 (31.3%)	21 (21.9 %)
Foot/ankle	31 (14.9%)	18 (18.8%)

The knee injury to the ligaments was the most frequent skiing injury. Of all skiing injuries, 25% were knee ligament damage (n=52/208). Knee ligament injuries were followed by ankle sprain (8.6%) and clavicle fractures (6.7%). In injuries occurring during snowboarding, distal radius/ulna fractures (n=16/96) ranked first (16.6%). These injuries were followed by clavicle fractures (12.5%) and ankle sprains in those injured during snowboarding (10.6%). Skier's thumb, which refers to acute injuries to the ulnar collateral ligament (UCL) of the thumb, defined in skiers, was observed in five patients (2.4%). The condition known as "snowboarder's fracture," which refers to talus lateral process fracture, was observed in three patients (3.1%) who had engaged in snowboarding activities. The five most common extremity injuries in patients skiing and snowboarding are shown in Table 4.

**Table 4:** Five most common extremity injuries in patients skiing and snowboarding. ACL: anterior cruciate ligament

Skiing (n, %)		Snowboarding (n, %)	
ACL injury	31 (14.9%)	Distal radius/ulna fracture	16 (16.7%)
Knee ligamentous injury (without ACL)	21 (10.0%)	Clavicle fracture	12 (12.5%)
Ankle sprain	18 (8.7%)	Ankle sprain	10 (10.4%)
Shoulder sprain	14 (6.7%)	Knee ligamentous injury (without ACL)	9 (9.4%)
Malleolar fracture	12 (5.8%)	Shoulder sprain	8 (8.3%)

## Discussion

The most important finding of our study is the difference in the anatomical regions injured in skiers and snowboarders. While the most frequently injured anatomical region in skiers is around the knee (31.3%), the most commonly injured region in snowboarders is the hand-wrist region (23.9%). The injury proportion of anterior cruciate ligament (ACL) injuries (14.9%) was found to be higher among patients who sustained injuries while skiing, whereas distal radius/ulna fractures (16.6%) were more commonly observed among individuals engaged in snowboarding activities. Furthermore, the study sample consisted predominantly of patients classified as being at the beginner or pre-intermediate skill levels.

The winter sports of skiing and snowboarding are increasingly gaining popularity among young adults, though with a significant association with consequential orthopedic injuries [5,9]. While our study did not encompass individuals below the age of 18, the composition of our study cohort, which primarily comprised a youthful demographic with 60% of patients self-reporting as a beginner or intermediate level, implies a growing prevalence of these sports. Consequently, it is likely that injuries will persist as a concern among the younger population.

According to the self-reports of the patients in our study group, more than half (60.8%) were classified as being at the beginner-intermediate level. The findings of our study are consistent with prior research and a meta-analysis, which have identified lower ability as a significant risk factor for injuries [1,10-12]. A comparative analysis examining the correlation between objective skill level and self-reported ability revealed that individuals who engage in skiing and snowboarding tend to overestimate their proficiency level [12]. This finding suggests that a higher proportion of injured participants may be beginners, as opposed to what they self-report. The task of educating novice skiers and snowboarders about ski resort safety and promoting responsible skiing or boarding within one's abilities continues to be a significant and complex undertaking for ski resorts.

While both skiing and snowboarding carry the potential for injuries, there exist notable differences in the anatomical distribution of the injuries incurred. The existing body of literature encompasses studies that investigate the anatomical disparities associated with injuries sustained during skiing and snowboarding activities. Both skiers and snowboarders are susceptible to lower extremity injuries, although skiing has a higher overall incidence of such injuries compared to snowboarding [5,9,13]. On the contrary, snowboarders exhibit a higher incidence of upper extremity injuries compared to skiers. Snowboarding participants exhibit greater susceptibility to wrist, shoulder, and clavicle injuries in comparison to skiers [9,13,14]. When the entire patient group included in our study was examined, it was determined that injuries affecting the lower extremities constituted approximately 53% of the total injury cases. In addition, similar to the literature results, it was determined that the majority (56%) of injuries occurring during skiing activities affected the lower extremities, and approximately 45% of the injuries that occurred during snowboarding activities were lower extremity injuries.

In the realm of snow sports, injuries to the shoulder girdle are prevalent, with a higher incidence observed among snowboarders compared to skiers [6]. Shoulder injuries predominantly arise from falls during skiing or snowboarding activities. In their study, Kim et al. investigated the incidence of snowboarding and skiing injuries at a resort in Vermont over a period spanning from 1988 to 2006. The researchers observed that shoulder injuries and clavicle fractures were prevalent among adult snowboarders, constituting 11.7% and 4% of the total reported injuries, respectively [9]. McCall and Safran conducted a study that revealed that shoulder girdle injuries constitute a range of 4% to 11% of total injuries incurred during downhill skiing. Among these injuries, rotator cuff strains were identified as the prevailing shoulder injury [15]. The findings of our study revealed that clavicle fractures constituted 6.7% of skiing injuries and 12.5% of snowboarding injuries. In a retrospective study conducted by Ogawa et al., the authors examined cases of glenohumeral dislocations in snowboarders and skiers who received treatment at a hospital in Japan for five years [16]. Among skiers, glenohumeral dislocations constituted 5.5% of the total injury cases. Our study found that the rates of glenohumeral dislocation were 2.8% and 3.1% among participants engaged in skiing and snowboarding activities, respectively.

Although injuries around the wrist are common injuries in both skiers and snowboarders, it is known that they are more common during snowboarding activities [6,14]. In our study, injuries around the wrist accounted for approximately 15% of all injuries among both skiers and snowboarders. Fractures of the distal radius and ulna constituted 31.4% of upper extremity injuries observed in snowboarding activities. The contact between the hands and the ground during a backward fall (i.e., heel side) is believed to be a crucial self-protective mechanism contributing significantly to wrist injuries.

Skier's thumb is a term used to describe an acute injury to the UCL that occurs when the thumb is hyperabducted or hyperextended during contact with the ground [17]. In their study investigating hand injuries in skiers, Keramidas et al. found that UCL injuries accounted for 19.4% of all hand injuries [18]. By contrast, it was found that UCL injuries constituted a mere 1.8% of the total reported hand injuries observed in snowboarders [14]. In our study, we also identified the occurrence of Skier's thumb lesions in a sample of five individuals who engaged in skiing activities. This constituted 23.8% of hand injuries observed in individuals engaged in skiing activities. However, the presence of these lesions was not observed in any individuals who participated in snowboarding activities.

In the literature, the most frequently observed injuries when assessing lower extremity injuries are sprains and ligamentous injuries in the knee region [6,8,9,12,13]. Kim et al. observed that skiing injuries involving the ACL accounted for 17.2% of all skiing injuries, whereas snowboarding injuries constituted only 1.7% of the total injuries [9]. Bere et al. identified three primary mechanisms of injuries, namely, back-weighted landing, slip-catching, and dynamic snow plowing. The primary factor contributing to the malfunction of the dynamic snow plowing and slip-catching mechanisms was the occurrence of knee internal rotation and/or valgus loading [19]. While ACL injuries are less prevalent among snowboarders, one hypothesized mechanism involves eccentric quadriceps contraction following the landing on a level surface with a flexed knee. This contraction leads to a forced internal rotation of the knee, potentially resulting in injuries to the ACL [20]. The findings of our research indicate that ACL injuries account for 14.9% of the total injuries observed in skiing, while snowboarding is associated with a lower prevalence of such injuries, specifically 3.1%.

The introduction of enhanced ski boots and bindings has been associated with a decrease in foot and ankle injuries among individuals engaged in skiing activities [6]. Although the overall incidence of lower extremity injuries is higher in skiers, injuries of the foot and ankle are more common in snowboarders, with foot and ankle injuries representing approximately 15% of all injuries in snowboarders [20]. In the present study, ankle injuries comprised 14.9% of all extremity injuries among skiers, whereas snowboarders exhibited a higher proportion of 18.7% for such injuries. Ankle sprain was the most common ankle injury in both snowboarders and skiers, and it was detected in 8.6% of skiers and 10.4% of snowboarders.

A specific type of injury that is commonly associated with snowboarding is a fracture of the lateral talar process, which is informally known as the snowboarder's fracture. Snowboarder's fracture typically arises due to a rapid increase in ankle dorsiflexion, accompanied by hindfoot inversion and axial loading. The occurrence of lateral process talus fractures is relatively infrequent, constituting approximately 1.2% to 6.3% of all lower extremity injuries observed in individuals engaged in snowboarding activities [21]. By contrast, Kim et al. reported in their study that talus lateral process fractures account for a mere 0.2% of the total snowboard-related injuries [9]. The present study revealed that 3.1% of snowboard-related injuries were identified as talus lateral process fractures.

In our study, we also encountered the most common knee ligament injury in skiers and the most common distal radius/ulna fractures in snowboarders. Most of the injuries were caused by individual falls, not collisions. For this reason, we think that the use of personal protective equipment specially produced for these frequently injured areas should be encouraged.

Although our study is the first study in our country on recreational skiing and snowboarding injuries, it has various limitations. First, our study is of a retrospective nature. Second, the present study assessed data obtained from a single center, which may possess limitations in its ability to identify a diverse spectrum of injuries comprehensively. Furthermore, an accurate count of the individuals who have submitted applications to the ski resort to which our data pertains has yet to be procured. Hence, it was unfeasible to provide a conclusive incidence and prevalence outcome.

## Conclusions

The popularity of recreational skiing and snowboarding has experienced a notable increase in recent times. There are distinct variations in the anatomical regions that sustain injuries during skiing and snowboarding activities. While individuals with limited experience are at a higher risk of sustaining injuries, no statistically significant disparity was observed in terms of the specific body parts affected among individuals of varying skill levels.

The recognition of these sports-related injuries, the prevalence of which is increasing in the young population, will facilitate the use of personal protective equipment to help prevent injuries, ensure environmental safety in ski resorts, and prevent morbidities related to these sports.
